# Real-World Evidence: Better than Nothing or the Future? A Perspective on Clinical Evidence Generation in Interventional Oncology

**DOI:** 10.1007/s00270-025-04179-4

**Published:** 2025-09-25

**Authors:** Nathalie C. Kaufmann, Philippe L. Pereira

**Affiliations:** 1 Cardiovascular and Interventional Radiological Society of Europe, Neutorgasse 9/6, 1010 Vienna, Austria; 2 Next Research GmbH, Neutorgasse 9/4a, 1010 Vienna, Austria; 3 Center for Radiology, Minimally-Invasive Therapies and Nuclear Medicine, SLK Kliniken Heilbronn GmbH, University of Heidelberg, Am Gesundbrunnen 20-26, 74078 Heilbronn, Germany; 4 University of Tübingen, Am Schnarrenberg, 72076 Tübingen, Germany; 5 Danube Private University (DPU), Steiner Landstraße 124, 3500 Krems an Der Donau, Austria

**Keywords:** Registries, Real-world data, Real-world evidence, Randomised controlled trials, Interventional radiology, Oncology, Observational studies

## Abstract

Randomised controlled trials (RCTs) have traditionally been regarded as the gold standard in evidence-based medicine, providing a high level of evidence. However, mounting complexities, ranging from ethical issues and logistical barriers to rapid technological evolutions, have sparked interest in alternative or complementary evidence-generation methods. Real-world evidence (RWE) from large, well-designed research projects is increasingly viewed as powerful tools. Although RWE is sometimes perceived as “less robust” compared to RCTs due to potential biases, recent advances in study design, data capture, and analytical frameworks have highlighted its potential for shaping clinical practice and informing regulatory decisions. This article discusses the benefits and challenges of RWE within interventional oncology and explores whether it is merely “better than nothing” or indeed represents a key pillar of the future of clinical research.

## Introduction

The introduction of interventional oncology into the mainstream of cancer care over the last two decades has spurred a strong demand for robust clinical evidence. Procedures such as thermal ablation, radio- and chemoembolisation, and other innovative image-guided therapies have become increasingly commonplace in oncology practice, offering minimally invasive options for cancer treatment. However, these therapies evolve rapidly: technologies improve, techniques change, and new adjunct therapies emerge with striking frequency. Evidence generation in this rapidly changing field, therefore, poses a challenge. Furthermore, while common malignancies such as hepatocellular carcinoma (HCC) involve enough patients to support large RCTs, many other IO interventions are performed in relatively small patient populations, making adequately powered RCTs infeasible. In such cases, systematically pooling data through multi-centre registries helps combine patients from several institutions to strengthen the evidence base when trials cannot be conducted. As a result, regulators, payers, and clinicians alike are increasingly focused on demonstrating meaningful improvements in survival, quality of life, and cost-effectiveness by real-world evidence [1].

Traditionally, randomised controlled trials (RCTs) are regarded as the pinnacle of evidence-based medicine, providing the highest level of internal validity due to their capacity for minimising bias through randomisation [2–4]. However, designing and conducting RCTs for interventional oncology’s locoregional treatment modalities can be extraordinarily difficult [5].As Buyse et al. (2011) note, survival endpoints may be confounded by the introduction of novel systemic therapies or combination regimens and retreatments. Furthermore, real-world complexities, such as heterogeneous patient populations, varied treatment settings, and decentralised care delivery, may not be adequately reflected in tightly controlled RCT environments [7, 8]. These shortcomings often diminish the external validity of even the most meticulously designed trials [9]. It is not surprising that although radiologic RCTs have expanded in quantity over the last 20 years, consistent methodological rigour remains a challenge [10]. For example, two 2009 sham-controlled RCTs of vertebroplasty concluded that the procedure offered no benefit [11, 12], leading to its widespread abandonment; over a decade later, a more targeted trial demonstrated significant pain relief with vertebroplasty in appropriate patients [13], illustrating how flawed RCT findings can misguide clinical practice.

Given these challenges, there is growing appreciation for the role of real-world evidence (RWE) in interventional oncology that captures data from broader populations, often in near real time [14]. RWE—systematic collections of real-world data—are becoming a cornerstone of this approach. In this article, RWE refers to evidence derived from real-world data sources, such as patient registries and well-designed single-arm observational studies, as opposed to evidence from randomised trials. Common study designs contributing to RWE include cohort studies (both retrospective and prospective), case–control studies, and pragmatic trials. While many of these are retrospective in nature, increasingly they are prospectively designed to enhance data quality and clinical relevance. These approaches allow the evaluation of device safety, procedural outcomes, and patient-centred measures by enrolling large numbers of patients under routine clinical care conditions [15–17]. Such real-world data (RWD) can be converted into real-world evidence (RWE), serving an increasingly pivotal role in decision-making for physicians, regulatory bodies, and payers alike. In line with this, much of the evidence that resulted in the inclusion of IO treatments in clinical practice guidelines originates from RWE rather than from RCTs [5]. Even for early-stage curative treatments, the inclusion of IO in guidelines has not been based on RCT data [18].

Against this backdrop, this article discusses the role of RWE in interventional oncology and its potential to complement or even, in specific contexts, supplant some aspects of the RCT model. By examining both the promises and pitfalls of this real-world approach, this manuscript seeks to answer the provocative question: **Is real-world evidence merely a compromise when RCTs are not feasible, or does it offer a glimpse into the future of clinical research?** Additionally, it outlines best practices for RWE data collection and provides examples of RWE that have influenced medical practice in interventional radiology/oncology. In doing so, it aims to illuminate how RWE might not only bridge current evidence gaps but also catalyse rapid, patient-centred innovation in a field that urgently needs it.

## Traditional Randomised Controlled Trials vs Real-World Evidence

While RCTs have long been established as the gold standard in clinical research, providing the highest level of evidence together with meta-analyses [19, 20]. Setting up and conducting a large-scale clinical trial is a lengthy and expensive endeavour [6, 21, 22]. Following protocol design and ethical review processes, the time from patient accrual to publication of oncology trials has been reported to be as high as 47 months [23]. Despite this long timeframe, the quality and utility of RCT data is frequently dampened by follow-up timelines that are too short to draw meaningful conclusions [20].

However, designing and executing classical RCTs in interventional oncology is often thwarted by a confluence of practical challenges. For example, technology and techniques may evolve faster than a trial can be designed, conducted, and analysed; protracted timelines can lead to findings that are outdated by the time they are published, potentially rendering results less relevant to current practice. Additionally, IR therapies are frequently combined with systemic treatments (e.g. chemoembolisation alongside chemotherapy), complicating trial designs and blurring attribution of outcomes. Second, RCTs generally employ strict eligibility criteria to ensure internal validity [24]. While this strict patient selection helps minimise confounding variables, it often excludes patients with comorbidities, advanced disease states, or less common tumour types. As a result, it can be difficult to generalise results of RCTs to the broader population routinely seen in clinical practice [9]. An additional layer of complexity in IO is variations in technique between operators and centres (i.e. lack of standardisation in how IR procedures are performed) can introduce heterogeneity that is difficult to control. Furthermore, the extent to which progression-free survival as a study endpoint and target effect size are utilised within oncology trials has been questioned regarding its provision of meaningful benefit to patients [25]. Finally, patient referral patterns can impede enrolment; many candidates for IR interventions are never referred to an interventional radiologist due to factors like limited awareness or competition with surgical specialties, making it challenging to recruit adequate patient cohorts for randomisation. Together, these factors help explain why relatively few high-quality RCTs exist in interventional oncology and why alternative evidence pathways are often necessary.

In contrast to RCTs (see Table 1), RWE capture the breadth of real-world clinical scenarios. By collecting RWD that reflect routine practice from a variety of sites rather than expert centres only, these approaches allow for the inclusion of more diverse patient populations [15, 26]. While patient selection bias is generally lower in real-world studies, self-selection through voluntary data submission as well as physician– patient selection cannot be excluded. As a result, patients who end up in real-world studies may differ systematically from those enrolled in RCTs or those who decline participation altogether.

**Table 1:** Comparison of key characteristics of RCTs and RWE [9, 14, 17, 20, 24]

	RCT	RWE
Cost-effectiveness	low	high
Timeliness	low	high
Generalisability	low	high
Flexibility	low	high
Longitudinal Safety Surveillance	low	high
Incomplete or inconsistent data	low	high
Selection Bias	potentially high	potentially high
Causal Inference	high	low
Cost	high	low to high

Unlike RCTs, where randomisation helps delineate cause-and-effect relationships, observational studies often struggle with residual confounding. While causality can sometimes be inferred through robust methodology, it is never as clear-cut as in a well-controlled trial. However, advancements in statistical and methodological techniques, such as propensity score matching, instrumental variable analysis, and sophisticated data analytics, are narrowing the gap between traditional RCT findings and real-world data [3, 4, 27]. For example, multivariable regression analysis remains a cornerstone for adjusting known confounding factors and estimating associations in observational datasets. Propensity score-based methods (e.g. matching, stratification, inverse probability weighting) further improve causal inference by balancing covariates between treated and control groups, effectively simulating the conditions of a randomised experiment and reducing bias in effect estimates [28]. Furthermore, modern machine learning algorithms are increasingly deployed to leverage high-dimensional, real-world datasets—these techniques excel at pattern recognition and outcome prediction, and when combined with advanced causal inference frameworks (such as targeted learning approaches) they can assist in estimating treatment effects more reliably from observational data [29]. By employing such tools, researchers can better mitigate residual confounding and extract credible real-world evidence that more closely approaches the clarity of RCT results.

Moreover, RWE offers a more flexible approach to data capture, often facilitating near real-time analysis of device performance, procedural outcomes, and patient-centred measures [17]. Regulatory authorities and professional societies increasingly mandate post-marketing registries to monitor long-term outcomes and detect rare adverse events [30–32], reflecting the growing belief that these observational data are critical for a more comprehensive picture of an interventional therapy’s safety and effectiveness. Overall, RWE already plays a significant and complementary role in the hierarchy of evidence for interventional oncology. Furthermore, by informing future RCTs and being strengthened through insights from RCTs, RWE is part of an evolving, circular relationship where each evidence source reinforces and refines the other.

It is worth noting; however, that no study, irrespective of its design, will yield reliable data unless it is designed, conducted, analysed, and reported in ways that are methodologically sound and appropriate to address the question it is hoping to answer. The quality of any study must be critically evaluated before its relevance to patient care can be considered [20].

## Examples of Impactful Real-World Evidence

Several high-profile registries highlight how real-world data can shape clinical practice and refine guidelines. Below are three examples that emphasise the impact of registry-based evidence across diverse medical specialties, including interventional oncology:

1. ARROW Trial (Oncology)

Although called a “trial,” the ARROW study incorporated a synthetic control arm constructed from observational real-world patient data to evaluate the efficacy of pralsetinib, a tyrosine kinase inhibitor, in non-small cell lung cancer (NSCLC). This design was particularly advantageous in targeting a relatively rare genetic alteration, allowing for an efficient assessment of the drug’s impact on small patient subgroups. By utilising observational data to supplement a smaller prospective cohort, the ARROW trial serves as a key example of how registries and real-world data can complement RCTs in oncology research [33, 34].

2. CRYODESMO-01 (Interventional oncology)

The prospective single-arm study CRYODESMO-01 in patients with desmoid tumours demonstrated an 86% one-year local control rate with cryoablation [35, 36]. In the absence of any RCT on this rare tumour type, this real-world study led to cryoablation being mentioned as a potential treatment in clinical practice guidelines [37, 38].

3. CIRT Registry (Interventional oncology)

The CIRT registry collected data on European patients undergoing radioembolisation of primary and secondary liver tumours. Its broad-based, real-world enrolment has provided nuanced insights into complication rates, long-term effectiveness, and overall patient outcomes, including quality of life. Given the complexity of organising large RCTs for relatively small patient subsets with secondary liver tumours, this registry’s real-world evidence has filled essential knowledge gaps and guided best practices [39, 40]. Looking ahead, a new European registry - CIEMAR (CIRSE Emprint Microwave Ablation Registry) - is prospectively collecting real-life data on microwave ablation of colorectal liver metastases, underscoring the field’s commitment to generating high-quality RWE in IO [41].

## Lessons from Pragmatic Prials and Hybrid Designs

Pragmatic clinical trials aim to assess interventions under usual care conditions, bridging the gap between the highly controlled methodology of RCTs and the broader applicability of observational studies [7, 8]. Interventional oncology stands to benefit significantly from the principles of pragmatic trial design, as these approaches allow for more flexible inclusion criteria and accommodate variations in clinical practice among different healthcare settings.

Efforts to establish a learning health care system guided by solid evidence have sparked calls to increase the number and improve the design of RCTs. One emerging avenue is to embed trials within health insurance systems, where insurance data can be used to both find eligible participants and assess outcomes. Although this approach remains relatively rare, it has already shown promise in several cases [42]. However, many hurdles remain to establishing trials within national health databases. In Italy, for example, electronic health records are not centrally and uniformly managed but are locally managed by 20 independent regions using differing technologies [43]. Once such hurdles are overcome, the ways in which health service provision is centrally recorded could open up new opportunities for randomisation. While not every patient population or intervention will fit this framework, stronger connections between insurance claims data and other datasets—alongside growing familiarity with the associated ethical and practical challenges—may broaden the scope and impact of these trials [22].

Increasingly, “hybrid” designs that incorporate randomisation within a real-world framework are being explored. In some cases, a registry enrols a large patient population, and a subset of these patients is randomised to different interventions [17]. This model offers several advantages:

- Enhanced internal validity: Randomising a subset of patients can help establish causality more firmly than purely observational data.
- Greater external validity: The broader registry still captures heterogeneous populations and evolving practice patterns, preserving the real-world context.
- Efficiency: Embedding an RCT within a registry capitalises on existing infrastructures for data collection, potentially reducing cost and time.

Such “registry-based RCTs” or “Trials within cohorts (TwiCs) have already been utilised in cardiology and orthopaedic surgery, with early successes in demonstrating not only clinical outcomes but also cost-effectiveness and patient satisfaction measures [44–46]. For interventional oncology, where iterative technical improvements and population heterogeneity are the norm, hybrid designs could offer a balanced approach. Real-world data from the registry provide a broad safety net for the detection of rare adverse events and long-term outcomes, while randomisation within a subgroup offers robust evidence for direct comparisons between competing interventions [47].

Where randomisation is not feasible, external control arms can be invaluable for enabling either formal or informal comparative analyses, thereby expanding how the results of an experimental arm are interpreted [48]. Such external controls can draw on RCTs, carefully curated real-world data sources (e.g. patient registries, electronic health records) or other observational datasets to represent standard-of-care outcomes. Regulatory agencies, including the FDA, have acknowledged the potential of external controls to bolster evidence for novel therapies, providing a pathway to accelerated approvals when robust randomisation is not possible [49, 50]. However, constructing a high-quality external arm requires rigorous patient selection, proper matching, and transparent data collection to minimise bias and ensure clinically meaningful conclusions [51]. When these methodological standards are met, external control arms can significantly reinforce the evidentiary base for emerging IO interventions.

In fact, multiple recent oncology trials have successfully integrated RWE-derived external control arms to complement or even replace a control group. In addition to the already mentioned ARROW study [33, 34], a single-arm study of mobocertinib for EGFR exon 20 insertion-mutated NSCLC compared its results against an external cohort drawn from electronic health records, demonstrating improved response rates and survival for the drug [52, 53]. These cases illustrate how carefully curated external comparators can yield credible insights and even support regulatory decisions, offering a pragmatic solution when standard RCTs are unfeasible.

Ultimately, pragmatic trials and hybrid designs underscore the notion that evidence generation should not be limited to a single methodological paradigm. By blending the rigour of randomisation with the real-world applicability of observational data, these approaches can yield higher-quality evidence that remains relevant to clinical practice and aligns with the rapidly evolving landscape of interventional oncology.

## Implications for Regulation and Policy

Regulatory authorities worldwide are increasingly recognising the value of RWE for approving new technologies and expanding indications for existing therapies. This is especially pertinent in interventional oncology, where devices may enter clinical practice through specialised pathways and require ongoing monitoring to ensure patient safety and effectiveness. Both the U.S. Food and Drug Administration and the European Parliament, for instance, have initiated programs and legislation specifically aimed at incorporating real-world data into the safety surveillance of medical devices [17, 32]. These efforts underscore a paradigm shift: instead of relying solely on pre-approval data, regulators now see the benefits of continuous, large-scale monitoring [16].

From a policy standpoint, the adoption of registry-based evidence can also affect reimbursement and health technology assessment [54]. Payers and health technology assessment bodies increasingly use real-world data to evaluate cost-effectiveness and budget impact, aiming to ensure that novel interventions deliver sufficient clinical benefits to justify their costs [1, 55]. High-quality real-world data, therefore, can expedite coverage decisions by demonstrating both effectiveness and economic value. Consequently, medical societies and advocacy groups have an opportunity to develop and maintain large registries, recognising their potential to streamline approvals and expand patient access to innovative therapies [31].

Nevertheless, the emerging reliance on real-world evidence also raises important questions about data governance, consistency, and the comparative rigour of RCTs. Policymakers must balance the need for comprehensive, flexible data sources against the risk of drawing conclusions from uncontrolled observations. Clear guidelines for data collection, reporting standards, and analytic methods are essential to ensure that registry-based evidence meets the regulatory rigour required for device approval and post-marketing surveillance. As the regulatory landscape evolves, well-designed and carefully maintained registries in interventional oncology could further manifest as a cornerstone for evidence generation, shaping both clinical practice and policy.

## The Future: A Symbiosis of Randomised Controlled Trials and Real-World Evidence

Given the inherent strengths and weaknesses of each model, the future of evidence generation in interventional oncology is likely to be neither exclusively RCT-driven nor purely observational. Instead, a synergy between the two approaches appears inevitable and indeed desirable. Traditional RCTs will continue to provide definitive evidence regarding efficacy and safety under controlled conditions, thereby maintaining their status as the gold standard for regulatory approval [21]. However, RWE will complement and enhance this evidence by capturing broader insights into real-world performance, patient-reported outcomes, and cost-effectiveness [15, 26]. At the same time, when a randomised trial is not feasible, due to limited patient numbers, ethical constraints, or even non-scientific (“political”) reasons, robust RWE can serve as an alternative to guide practice (for example, evidence from registries is often the only data available when evaluating ablation versus surgery for certain tumours).

Over time, such a dynamic interplay between RCTs and registries might transform interventional oncology research, shifting it away from a “snapshot” model of clinical evidence (dominated by isolated trials) towards a more fluid, evolving paradigm that keeps pace with technological and methodological advances.

## Improving Real-World Data Collection

To maximise the utility of RWE, improvements in standardisation across interventional oncology are paramount. Beyond standardisation of data collection, management, and endpoint selection, there is an urgent need for standardisation of treatment performance and follow-up [5].

Furthermore, the integration of electronic health records (EHRs) into registry data-collection systems could partially automate data capture, reducing the workload on clinicians and diminishing manual entry errors [1, 17]. Furthermore, emerging approaches that leverage artificial intelligence (AI) and machine learning (ML) can help clean and analyse large datasets by identifying anomalies, managing missing data, and generating predictive models [24].

Equally important is the governance of registry data. Frequent audits, transparent reporting of data quality metrics, and clear policies for data usage and sharing all contribute to maintaining the integrity of registry-based research [7]. In multi-centre or multinational registries, this may require complex frameworks that reconcile different privacy laws, ethical standards, and reporting requirements. However, as interventional oncology continues to expand globally, standardised governance frameworks could enable larger and more diverse registries that offer robust, generalisable findings.

Additionally, hybrid trial designs present new opportunities to improve internal validity within a registry context [26]. By randomising a subset of patients within a registry, researchers can maintain the benefits of real-world data collection while mitigating some of the limitations of purely observational designs[10, 24]. This approach provides a middle ground that leverages the naturalistic setting of registry data while incorporating at least some degree of randomisation.

## Conclusion

The dichotomy between rigorous RCTs and “lower tier” RWE is dissolving in the face of methodological advances and current needs for evidence, especially in a rapidly evolving field like IO. RWE offers a powerful means to gather real-world data that reflect diverse patient populations and treatment settings, often in near real time. By capturing safety signals, procedural outcomes, and patient-centred measures more quickly and cost-effectively than traditional randomised trials, registries provide an invaluable complement to the high internal validity of RCTs.

That said, RWE faces notable challenges, including the risk of selection bias, incomplete or inconsistent data, and limited ability to establish causality. Data analytics, pragmatic and prospective study designs, and robust governance frameworks can help mitigate these concerns, ensuring that the observational nature of registry-based evidence does not undermine its overall value. As regulators become more and more open to integrating real-world evidence into their decision-making processes, high-quality registries form the backbone of post-marketing surveillance and inform nuanced policy decisions regarding reimbursement and expanded indications.

Ultimately, interventional oncologists, regulators, and stakeholders will view RWE not as a replacement for RCTs but as an evolving paradigm that can better align clinical research with real-world practice. In this sense, well-constructed real-world studies are far more than “better than nothing”; they are a vital cornerstone of evidence generation in interventional oncology—one that informs best practices and ensures that patients reap the benefits of the latest technological and therapeutic advances. However, to maximise the scale and utility of real-world data in interventional oncology, there is a dire need for standardisation of clinical practice. By clearly delineating these benefits and challenges, this review may also aid in garnering support for establishing and maintaining the robust RWE needed to advance evidence generation in interventional oncology.
